# Pain Improvement With Novel Combination Analgesic Regimens (PAIN-CARE): Randomized Controlled Trial Protocol

**DOI:** 10.2196/resprot.7493

**Published:** 2017-06-08

**Authors:** Ian Gilron, Dongsheng Tu, Ronald Holden, Alan C Jackson, Nader Ghasemlou, Scott Duggan, Elizabeth Vandenkerkhof, Roumen Milev

**Affiliations:** ^1^ Queen's University Department of Anesthesiology and Perioperative Medicine Kingston, ON Canada; ^2^ Queen's University Kingston, ON Canada; ^3^ University of Manitoba Winnipeg, MB Canada

**Keywords:** neuropathic pain, alpha-lipoic acid, antioxidant, pregabalin, anticonvulsant

## Abstract

**Background:**

Neuropathic pain (NP) (including painful diabetic neuropathy, postherpetic neuralgia, etc) affects approximately 7% to 8% of the population and is associated with a devastating symptom burden as well as a profound economic impact for patients, their families, and the health care system. Current therapies have limited efficacy and dose-limiting adverse effects (AEs). Rational combination therapy with carefully selected NP drugs has shown potential for measurable improvements in pain relief, quality of life, and health care use. Today, over half of NP patients concurrently receive 2 or more analgesics but combination use is based on little evidence. Research is urgently needed to identify safer, more effective combinations.

**Objective:**

We hypothesize that analgesic combinations containing at least 1 nonsedating agent would be as safe but more effective than either monotherapy without increasing overall AEs because of additive pain relief. Pregabalin (PGB), a sedating anticonvulsant, is proven effective for NP; the antioxidant alpha-lipoic acid (ALA) is one of the only nonsedating systemic agents proven effective for NP. Thus, we will conduct a clinical trial to compare a PGB+ALA combination to each monotherapy for NP.

**Methods:**

Using a double-blind, double-dummy, crossover design, 54 adults with NP will be randomly allocated to 1 of 6 sequences of treatment with PGB, ALA and PGB+ALA combination. During each of 3 different treatment periods, participants will take 2 sets of capsules containing (1) ALA or placebo and (2) PGB or placebo for 31 days, followed by an 11-day taper/washout period. The primary outcome will be mean daily pain intensity (0-10) at maximally tolerated dose (MTD) during each period. Secondary outcomes, assessed at MTD, will include global improvement, adverse events, mood, and quality of life.

**Results:**

Participant recruitment is expected to begin September 1, 2017. The proposed trial was awarded external peer-reviewed funding by the Canadian Institutes of Health Research (Canada) on July 15, 2016.

**Conclusions:**

This trial will provide rigorous evidence comparing the efficacy of a PGB+ALA combination to PGB alone and ALA alone in the treatment of NP.

**Trial Registration:**

International Standard Randomized Controlled Trial Number ISRCTN14577546; http://www.isrctn.com/ISRCTN14577546?q=&filters=conditionCategory:Signs%20and%20Symptoms,trialStatus: Ongoing,recruitmentCountry:Canada&sort=&offset=1&totalResults=2&page=1&pageSize=10&searchType=basic-search (Archived by WebCite at http://www.webcitation.org/6qvHFDc6m)

## Introduction

Chronic pain has a prevalence of 20% to 25% [1] and is one of the most frequent reasons to seek health care and miss work [2]. Pain impairs physical, social, and occupational function and thus exerts a devastating impact on patients, their families, and society. Each year in North America, chronic pain costs over $650 billion in health care and lost productivity thus exceeding costs of heart disease, cancer, and diabetes [3]. Neuropathic pain (NP) is a common form of chronic pain caused by nervous system diseases [4,5] including radiculopathy, diabetic neuropathy, HIV-neuropathy, and cancer-related NP [6]. NP is more prevalent in the elderly and we need to prioritize treatment research as our population ages [7]. NP management involves treating underlying causes, reducing pain, and improving function. Systemic oral pharmacotherapy is a valuable component of multimodal NP management [8] given ease of administration and engagement of drug effect sites throughout the sensory nervous system. However, current treatments give only partial benefit due to incomplete efficacy and dose-limiting adverse effects (AEs) [8]. In addition to reversible AEs such as sedation, excessive pain-contingent dosing of drugs with incomplete efficacy can lead to catastrophic outcomes such as opioid-related [9] and anti-inflammatory drug (NSAID)–related toxicity and death [10].

Due to perceived benefits of combination therapy [11,12], more than 50% of NP patients concurrently receive 2 or more analgesics [13]. However, current combination prescribing is based on little evidence, some combinations may be harmful, and authorities have demanded more research to develop rational combination strategies [8]. This field has received little attention from industry, emphasizing the need for public funding. Rational combination therapy with mechanistically different agents has shown potential for measurable improvements in pain relief, quality of life, and health care utilization and, indirectly, fewer NSAID-related and opioid-related mortalities. Combination therapy has been studied in many therapeutic areas but only recently in NP. Although our previous trials [14-16] support the merits of combination therapy, other data indicate that some combinations confer no benefit in some conditions [17] and other combinations do more harm than good [18]. Our Cochrane review [11] identified only 21 NP trials of different combinations. This dearth of evidence emphasizes the urgent need for more research.

A combination of pregabalin (PGB, Lyrica) plus alpha-lipoic acid (ALA) is now the most important to study because (1) high-quality evidence supports the efficacy of these agents as monotherapies in NP; (2) ALA is inexpensive, widely accessible, and currently the only nonsedating systemic agent for NP [19]; (3) PGB also improves sleep, mood, and anxiety [20]; (4) PGB and ALA have complementary mechanisms providing the expectation of greater synergy; and (5) AE profiles are different and combining PGB+ALA will not increase overall AEs. Combining a sedating and nonsedating agent is a novel approach with vast potential for improved patient outcomes. This project directly addresses a desperate need to improve chronic pain treatment.

Both PGB and ALA are approved by Health Canada for clinical use and proven for the treatment of NP [8,19]. PGB blocks the α-2-δ subunit of N-type voltage gated calcium channels, resulting in decreased calcium influx and neurotransmitter release [21,22]. PGB is recommended as first-line treatment for NP [8], and a recent meta-analysis of 19 trials (7003 participants) yielded a number-needed-to-treat (NNT for 50% pain reduction) of 5.0 for NP [20]. AEs (at 600 mg per day) included sedation (15%-25%) and dizziness (27%-46%). Our experience with PGB includes a Pfizer-sponsored trial [23] and, more recently, an investigator-initiated trial of PGB plus duloxetine for fibromyalgia [24]. ALA has been extensively studied in NP, and its therapeutic effects in this setting appear to be, in part, due to its antioxidant actions [25]. In a rat model of streptozocin-induced diabetes, ALA delayed the onset of polyneuropathy [26]. Mechanistic studies suggest decreased nociceptive sensitivity by inhibition of T-type calcium (Cav3.2) channels [27], distinct from that of PGB, which inhibits N-type calcium channels [22], suggesting potential for synergy at these different sites of action. At least 16 trials of over 1320 patients have reported reductions in pain and other symptoms [19,28], and a recent meta-analysis reported an NNT of 6.3 [19]. Also, 1 trial reported improvement in NP symptoms after 4 years of treatment [29]. AEs of nausea, vomiting, headache, and vertigo have been reported in studies involving more than 1200 mg per day of ALA. There have also been rare reports of hypoglycemia (low blood sugar) in diabetic patients taking ALA and reporting symptoms of sweating, paleness, chills, headache, dizziness, and confusion. We identified only 1 study of a combination similar to PGB+ALA—ALA plus gabapentin (related to PGB) in the treatment of burning mouth syndrome [30]. Despite major methodological flaws, the study suggested greater benefit with this combination versus monotherapy, and AEs were reported overall as very mild [30].

Thus, our objective is to conduct an innovative double-blind, randomized controlled trial (RCT) to compare a combination of the anticonvulsant PGB with the nonsedating antioxidant ALA to each monotherapy in NP.

## Methods

### Ethics

This study underwent ethics review and received a compliance notice by the Queen’s University Health Sciences and Affiliated Teaching Hospitals Research Ethics Board on December 15, 2016. This trial will be conducted at one site, Providence Care Centre, Kingston, Ontario, Canada.

### Aims and Hypothesis

The objective of this trial is to compare the safety and efficacy of a PGB+ALA combination to each monotherapy in treating participants with NP. Our primary hypothesis is that PGB+ALA has greater analgesic efficacy versus either monotherapy.

### Trial Design

**Figure 1:**
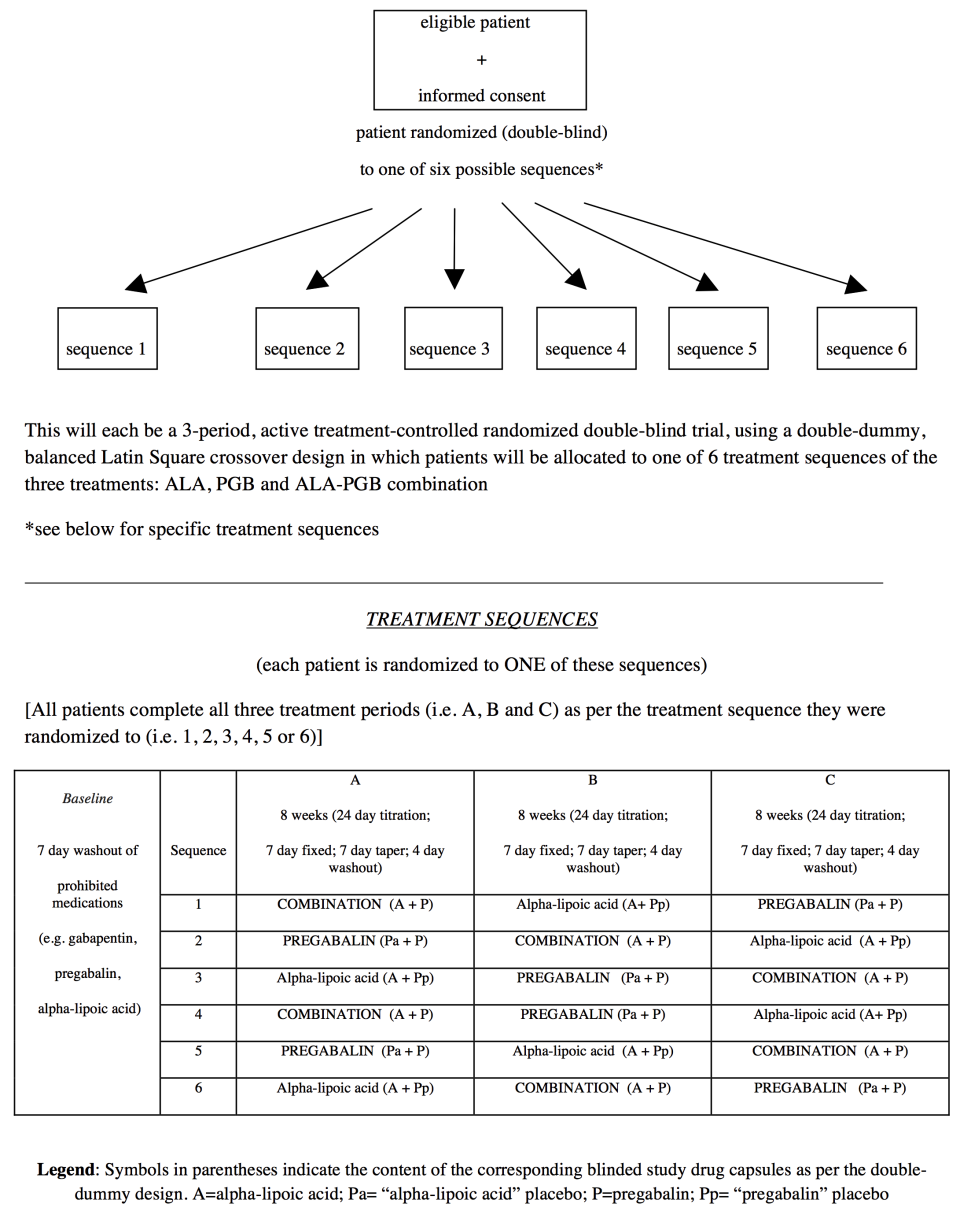
Trial design.

**Figure 2:**
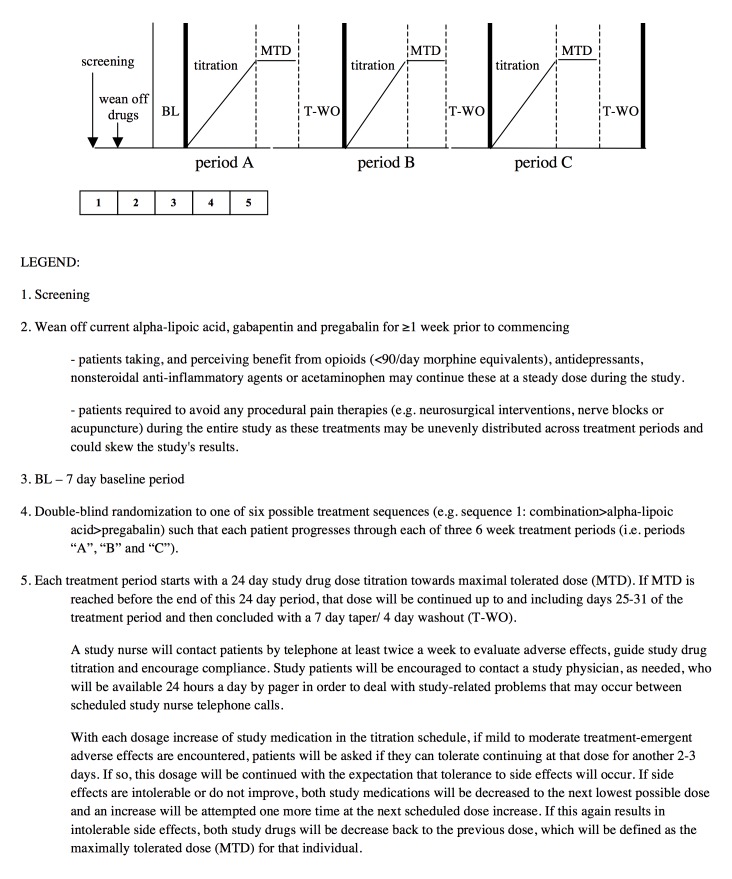
Trial design, continued.

We have designed a single center double-blind, double-dummy, randomized, controlled, 3-period crossover trial (compliant with Health Canada; International Conference on Harmonization; Methods, Measurement, and Pain Assessment in Clinical Trials; and Consolidated Standards of Reporting Trials [CONSORT]) comparing a PGB+ALA combination to monotherapy in treating NP (Figures 1 and 2). Using a flexible dose titration, Latin Square crossover design, treatments will be titrated during each of 3 treatment periods to maximal tolerated dose (MTD) with primary and secondary trial analyses comparing the 3 treatments using end-of-period outcomes. Given ethical issues and since PGB and ALA are proven with multiple trials confirming superiority over placebo [19,20], this active control superiority design does not include a placebo alone treatment. Furthermore, our previous trials [14-16] have confirmed assay sensitivity with a similar design. Internal validity of our crossover design is supported by stability of NP over time [14-17,31] and the risk of carryover from one period to the next is very low because each period is followed by an 11-day dose taper and drug washout, and the final MTD week for each period (from which the primary outcome is obtained) is separated from the next period’s final week by 7 weeks (ie, ≥20 half-lives of the drugs studied). Nevertheless, exploratory analyses will be conducted to identify if any low-order carryover effect does exist.

**Figure 3:**
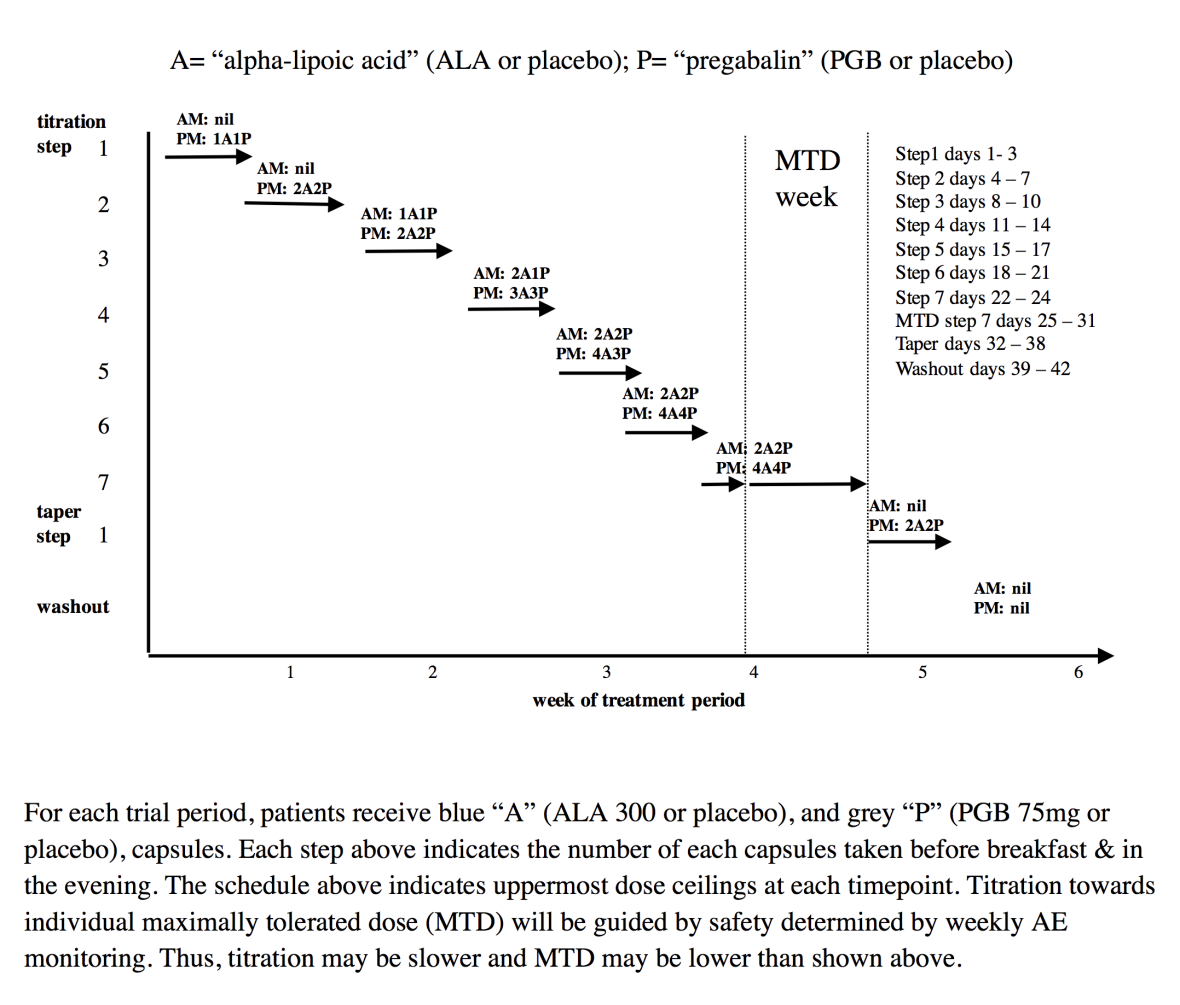
Study drug schedules.

During each of 3 trial periods, using a double-blind randomized crossover design, patients will receive 2 sets of capsules (Figure 3): (1) blue capsules (ALA 300 mg or placebo) and (2) gray capsules (PGB 75 mg or placebo). During the combination period, blue will contain ALA and gray will contain PGB. During the ALA alone period, blue will contain ALA and gray will contain placebo. During the PGB alone period, blue will contain placebo and gray will contain PGB.

Consenting patients on ALA or PGB (or gabapentin) pretrial will agree to be weaned gradually for a washout of at least 7 days. Patients taking and perceiving benefit from opioids (<90 mg morphine equivalents), antidepressants (tricyclic, selective serotonin reuptake inhibitor, or serotonin–norepinephrine reuptake inhibitor), NSAIDs, or acetaminophen may continue these at a steady dose for the entire study. Any cognitive behavioral therapy or exercise programs may continue only if they can be scheduled evenly across all treatment periods. Research staff will monitor and advise patients weekly about prohibited cointerventions throughout the study. A thorough understanding of the threats to validity of using forbidden cointerventions is heavily emphasized to participants. Patients will not be allowed to start new cognitive behavioral therapy or exercise programs after study initiation and must avoid any procedural therapies (eg, nerve blocks or acupuncture) during the entire study. Any pain exacerbations that in the opinion of the patient warrant initiation of a new therapy would necessitate trial discontinuation and immediate weaning from study medications, but these patients would still be included in the trial analyses.

### Dose Titration

Study medication will follow a flexible dose titration to MTD to balance tolerability and relief, with regular weekly calls by research personnel. This means that doses of study medication will not be further increased if intolerable AEs are encountered at higher doses or if “a lot” or “complete” pain relief is achieved. If AEs are experienced subsequent to a dose increase of study medications, the protocol will allow for dose reduction to the previous dosage level in order to improve tolerability. The MTD fixed dose week will be from days 25 to 31. However, if MTD is reached before day 25, that MTD dose will continue up to and including the day 25 to 31 period. For ALA, the maximum possible daily dose will be 1800 mg, and for PGB the maximum possible daily dose will be 450 mg. The MTD fixed dose week will be followed by a 7-day dose taper and 4-day complete washout. Daily pain ratings will be completed throughout the trial. During dose taper and washout periods only, patients may take acetaminophen, ≤8 tablets per day (325 mg per tablet) as needed. This rescue medication is very unlikely to affect the primary outcome measure of pain intensity during the MTD phase of each treatment period.

### Participant Allocation

As per the 3-period Latin Square crossover, patients are randomly allocated to 1 of 6 sequences of ALA, PGB, and combination (Figure 1). Before the trial, an independent pharmacist and biostatistician will prepare a concealed allocation schedule using a computer-generated block randomization process to randomly assign treatment sequences to a consecutive series of numbers within a block. Each patient will be assigned to the next consecutive number, and the corresponding sequence of medications will be dispensed. All study personnel will be blinded to the block sizes to preserve allocation concealment.

### Protecting Against Bias

Medications will be encapsulated (ALA, blue; PGB, gray) in an identical fashion across all periods. As per a double-blind, double-dummy design, patients will take both sets of medications so all treatment conditions will be identical across all 3 treatment periods. Treatment codes will be generated by the investigational pharmacist and concealed until trial completion. In case of emergency, individual codes will be disclosed by an investigational pharmacist to a nonstudy clinician. A questionnaire completed by every participant at the end of each period will ask patients to guess the treatment they received to assess blinding.

### Inclusion and Exclusion Criteria

With the input of longstanding specialist and primary care colleagues in the Kingston and Queen’s University catchment area, men and women meeting the diagnostic criteria for peripheral NP will be considered for the trial. Participants must have a score of 3 or higher on the Douleur Neuropathique 4 interview, a validated questionnaire that distinguishes between neuropathic and nonneuropathic pain [32]. As indicated, investigations will be done to confirm NP diagnosis including, but not limited to, nerve conduction studies and electromyography. After preliminary phone screening, candidates will be invited to the clinic for a detailed history, physical, neurological examination, and a review of recent (within the last 3 months) lab studies including complete blood count, glucose, electrolytes, urea, creatinine, aspartate transaminase and alanine transaminase (AST/ALT), glycosylated hemoglobin, and electrocardiogram. Laboratory analysis will be conducted for study candidates with no recent records. Eligible patients will have daily pain (≥3/10) for at least 3 months, AST/ALT ≤120% upper limit of normal, creatinine clearance ≥60 mL per minute, and glycosylated hemoglobin ≤9.5%. Patients will have necessary abilities, visual acuity, and language skills for questionnaire completion and phone communication with nurses. Patients with major organ system disease, cardiovascular autonomic neuropathy, moderate to severe sedation or ataxia due to other required drugs, hypersensitivity to study medications, seizure disorder, or other painful condition >50% as severe as their NP will be excluded. Patients with a major, poorly controlled psychiatric disorder, severe depression or suicidal ideation, or active substance abuse disorder will be excluded. Patients with a history of angioedema will be excluded. Those who live alone and cannot assure daily contact with a friend, family member, or caregiver will be excluded. Women of childbearing potential will be required to receive a highly effective form of contraception (total abstinence, hormonal birth control methods, intrauterine devices, confirmed successful vasectomy of partner, double barrier methods, etc) and a negative pregnancy test at baseline. If a study participant becomes pregnant, she must stop using study medications immediately and will be withdrawn from the study. Eligible patients will be enrolled into the study following informed consent.

### Trial Duration and Follow-Up Frequency

**Figure 4:**
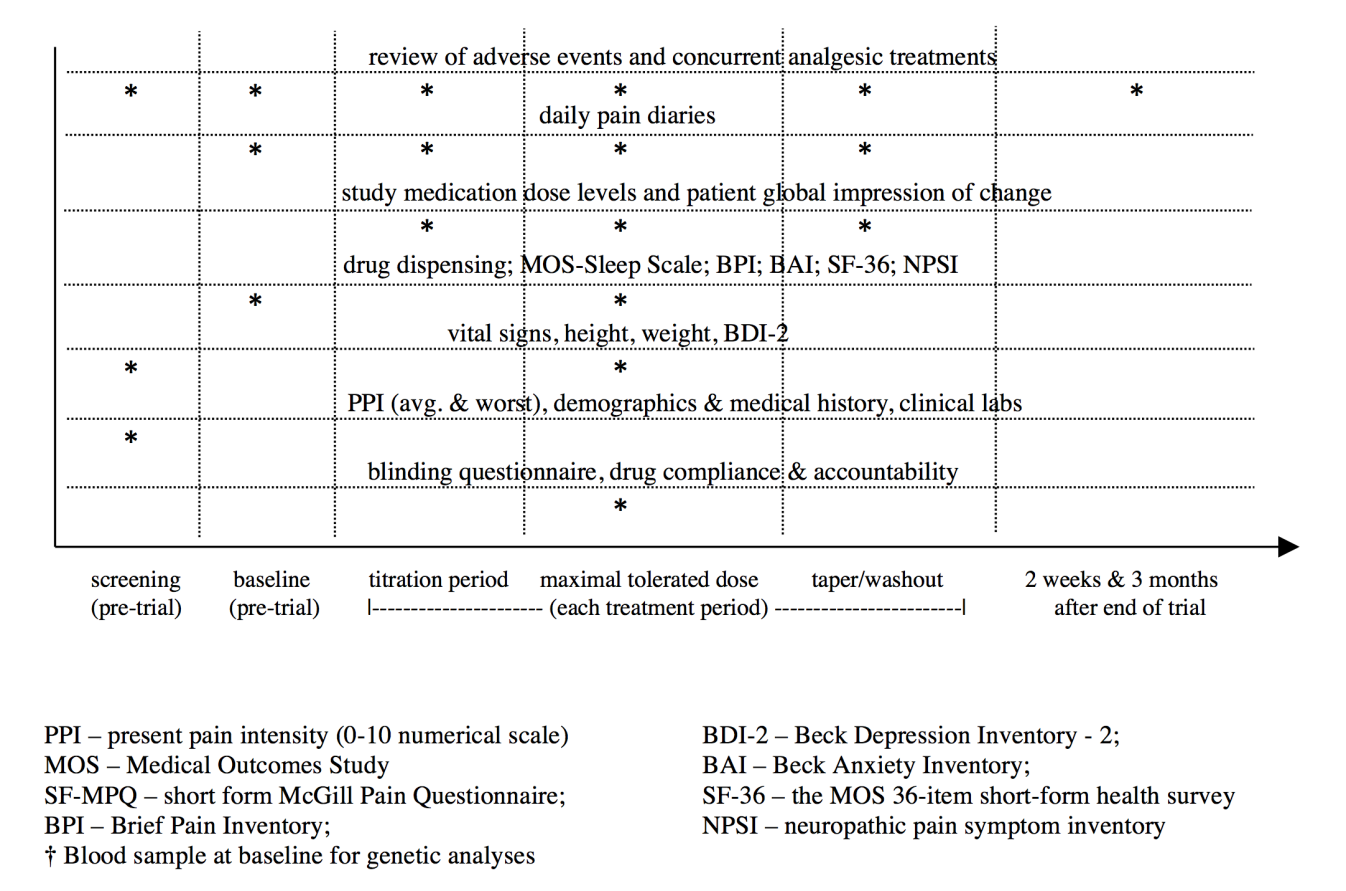
Schedule of assessments.

Each of the 3 treatment periods will be 6 weeks, for a total trial duration of 18 weeks. The nurse will phone patients weekly to evaluate AEs, guide drug titration, and encourage compliance. Patients will be seen in the clinic at the end of each treatment period for assessment of vital signs and measurement of secondary outcomes (Figure 4). Patients will be followed up by phone 2 weeks and 3 months after trial completion (including patients who were withdrawn from the trial prematurely) to document any subsequent AEs.

### Outcome Measures and Safety Assessment

The primary outcome is mean daily pain (0-10 numerical rating scale with 0 = no pain, 10 = worst pain imaginable) averaged over the MTD fixed dose week (days 25-31) of each period (Figure 4). Secondary outcomes include daily pain at other time points, the MTDs of PGB and ALA, frequency and severity of AEs and global relief, the short form McGill Pain Questionnaire [33], the Neuropathic Pain Symptom Inventory [34], the Brief Pain Inventory [35], the Beck Depression Inventory-II [36], Beck Anxiety Inventory [37], the Short Form survey (SF-36) [38], blinding questionnaires, and acetaminophen consumption. Timing of outcome assessments is described in Figure 4. Patient safety will be ensured by vigilant AE assessment and judicious drug titration. Any occurrences of major AEs will be tracked as secondary outcomes and also reported to the Queen’s Ethics Board, Health Canada. Assessment and reporting of AEs will adhere to CONSORT recommendations [39].

### Sample Size

Based on previous estimates of within-patient variation, variance=2.3, from our previous study in NP [15], we calculate that a sample of 55 trial completers would provide an 80% chance of detecting (alpha=0.05) a mean treatment difference of 1 point (0-10 scale). For a sample size divisible by 6, the number of treatment sequences, we adjusted the sample size to 54 trial completers.

### Statistical Analyses

Analyses for this trial are based on the null hypothesis of no difference between PGB, ALA, and PGB+ALA and the alternative hypothesis that at least 2 treatments are different. When a patient contributes data from only one period, sensitivity analyses including all patients will also be performed by assuming some reasonable but extreme values for the remaining periods. All patients receiving at least one dose of drug will be included in the safety analyses.

The primary outcome—mean daily pain from the last 7 days (at MTD) of each treatment period—will be calculated as an average of pain scores as recorded in the pain diary if more than 50% of the information (ie, at least 4 days) is not missing. Otherwise, mean daily pain will be treated as missing. This is based on the half rule often used to summarize repeated responses and which has proven unlikely to introduce bias to trial results [40]. Sensitivity analyses based on the average of all available pain scores will also be performed to confirm the results of the primary analysis. Although carryover effects are unlikely, we recognize this possibility. Therefore, a linear mixed model with sequence, period, treatment, and the first order carryover term as fixed effects and patient as a random effect [41] will be used to test for differences among the 3 treatments and to estimate the least square mean of the mean daily pain intensity for each treatment, adjusting for carryover as well as period effects (ie, stability of pain levels). The following 3 pair-wise comparisons will be performed based on the least square means and standard deviations from the linear mixed model: combination versus ALA alone, combination versus PGB alone, and ALA alone versus PGB alone. Sensitivity analyses will be performed using a pattern-mixture model [42] based on patterns of missing data so as to check the robustness of results in the case that data may not be missing at random. A Fisher's least significant difference [43] procedure will be used to adjust the *P* values for these 3 comparisons.

Secondary outcomes will be analyzed similarly except that (1) only one measurement is analyzed in the last week for the singular measures (ie, final week questionnaires) and (2) the scoring algorithms developed for the Brief Pain Inventory, Beck Depression Inventory-II, and SF-36 will be first used to derive the subscales or domains within these instruments, and the scores on these subscales or domains will be used as response variables in the linear mixed model analysis.

## Results

Participant recruitment is expected to begin September 1, 2017. The proposed trial was awarded external peer-reviewed funding by the Canadian Institutes of Health Research (Canada) on July 15, 2016.

## Discussion

NP remains a challenging condition to treat, with current analgesic drugs providing only partial relief, often at the risk of disabling AEs. To the best of our knowledge, this proposed trial is the first to compare the combination of an anticonvulsant with an antioxidant to treat NP. Because ALA and PGB have different AE profiles, we expect their combination to provide superior analgesic efficacy in NP without increasing AEs.

Potential threats to completing this trial include challenges with participant enrollment, noncompliance, protocol violations, and early dropouts. However, we are confident that the proposed trial design and our experience with recent and previous RCTs will minimize these concerns. Also, noncompliance, protocol violations, and early dropouts will be minimized by the crossover design as well as thorough patient teaching and careful follow-up of trial patients.

As discussed above, there is an urgent need for improved NP treatments with better analgesic efficacy and better safety and tolerability. Thus, this trial shall provide rigorous evidence for a potentially improved treatment strategy for NP.

## Abbreviations

AE: adverse effect
ALA: alpha-lipoic acid
AST/ALT: aspartate transaminase and alanine transaminase
CONSORT: Consolidated Standards for Reporting Trials
MTD: maximal tolerated dose
NNT: number-needed-to-treat
NP: neuropathic pain
NSAID: nonsteroidal anti-inflammatory drug
PGB: pregabalin
RCT: randomized controlled trial
SF-36: Short Form survey
